# Foot Skin Ischemic Necrosis following Heel Prick in a Newborn

**DOI:** 10.1155/2013/912876

**Published:** 2013-10-28

**Authors:** Esad Koklu, Erdal Avni Ariguloglu, Selmin Koklu

**Affiliations:** ^1^Megapark Hospital, Department of Pediatrics, Division of Neonatology, Kahramanmaras, Turkey; ^2^Department of Obstetrics and Gynecology, Megapark Hospital, Kahramanmaras, Turkey; ^3^Turkey Ministry of Health, Necip Fazıl Hospital, Turkey

## Abstract

There are only a few reports on side effects after heel prick in neonates although heel prick has been performed all over the world for many years. The medicine staff had obtained only a drop of blood by pricking the baby's heel using a lancet without compressing the heel or foot to measure his blood glucose level 3 hours after birth. However he developed a severe and hemorrhagic skin reaction on his entire left foot, beginning 30 minutes after obtaining the drop of blood by pricking the baby's heel using a lancet. The lesion, which was treated with topical mupirocin and povidone-iodine solution daily, slowly decreased in size and had almost fully resolved within 3 weeks. He was healthy and 9 months old at the time of writing this paper. We herein report a case of foot skin ischemic necrosis following heel prick in a newborn. To our knowledge this patient is the first case of foot skin ischemic necrosis due to heel prick in newborns.

## 1. Introduction

Although heel prick has been performed all over the world for many years, there are only a few reports on side effects after heel prick in neonates. Severe local reactions including pain, swelling, tenderness, and erythematous plaques are well-known adverse effects of heel prick in newborn [1]. Skin bruising and necrosis during delivery, which are mostly attributed to birth trauma, may be the sign of underlying inherited thrombophilia [2]. However, to our knowledge, there has been no reported foot skin ischemic necrosis associated with heel prick in neonates. We herein report the first case of foot skin ischemic necrosis developing approximately 30 minutes after the heel prick in a newborn.

## 2. Case Report

**Figure 1:**
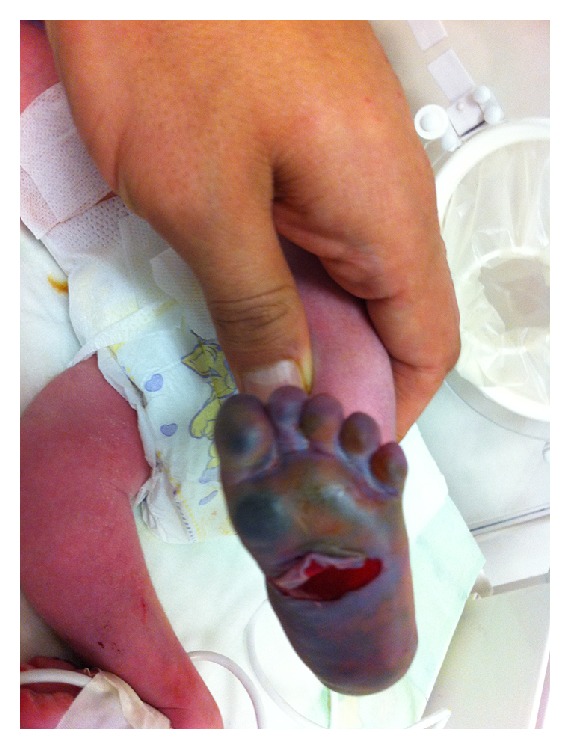
Hemorrhagic skin reaction and skin necrosis throughout his left foot.

The male infant was hospitalized at 4 hours of age owing to widespread cutaneous necrosis of his left foot (Figure 1). The medicine staff had obtained only a drop  of blood by pricking the baby's heel using a lancet without compressing the heel or foot to measure his blood glucose level 3 hours after birth. However he developed a severe and hemorrhagic skin reaction on his left entire foot, beginning 30 minutes after obtaining the drop of blood by pricking the baby's heel using a lancet. There was no history of umbilical catheterization and administration of parenteral drugs and hypertension, diabetes mellitus, renal diseases, lupus erythematosus, or thrombotic or hemorrhagic tendency in the mother, siblings, or other family members. Drug use during pregnancy was limited to routine antenatal drugs. Because of fetal distress at 38-week gestation, a newborn male was delivered by caesarean section to a 26-year-old multigravida mother. The parents were not relatives. The Apgar scores were 4 at 1 min and 9 at 5 min. No resuscitation was required. There was no dyspnoea. The chest X-ray was normal and blood gas analyses were within normal limits. His weight was 3160 g, length 49 cm, and head circumference 35 cm. Temperature was 36.2°C, respiratory rate 57/min, heart rate 158 beats/min, and blood pressure 65/43 mmHg. Sepsis workup was performed and the infant was commenced on ampicillin 50 mg/kg and amikacin 7.5 mg/kg twice a day, intravenously. Haemoglobin was 13.8 g/dL; leucocyte count 19 × 10^9^ cells/L with 59% polymorphonuclear cells, 37% lymphocytes, and 4% monocytes; and platelet count 243 × 10^9^/L. Color sonographic imaging of lower extremity was normal. Doppler cerebral ultrasound imaging was also normal. Coagulation studies were within normal ranges.

On examination, he had a severe necrotic and hemorrhagic skin reaction on his entire left foot (Figure 1). The affected area was initially well circumscribed and uniformly purple in color. Swelling and erythema appeared within a few hours. His left entire foot became bluish up to the groin and swollen at the age of 22 hours. Four days later the lesion developed a central area of dusky reddish gray color surrounded by a circle area of white yellow color and an outer peripheral area of erythema, respectively. The parents refused a skin biopsy on their infant. Six days later the lesion was described as a central area of black color surrounded by an area of yellow color. The lesion, which was treated with topical mupirocin and povidone-iodine solution daily, slowly decreased in size and had almost fully resolved within 3 weeks. He was healthy and 9 months old at the time of writing this paper.

## 3. Discussion

Localized skin necrosis of foot presenting after birth is unusual. Localized necrotic skin lesions must be considered as part of a systemic embolic process and can lead to extensive laboratory investigation and ultrasound exploration. However, laboratory investigation and ultrasound examinations were normal in our patient. Skin bruising and necrosis during delivery, which are mostly attributed to birth trauma, were not the cause of the presenting lesion in our case, because the baby had no hemorrhagic skin reaction on his left foot at birth. There was no tourniquet or hand squeezing for the left leg and foot. However he developed a severe and hemorrhagic skin reaction on his entire left foot, beginning 30 minutes after obtaining the drop of blood by pricking the baby's heel using a lancet 3.5 hours after birth.

The parents refused a skin biopsy on their infant and the true etiology of the lesion could not be demonstrated. Skin necrosis during delivery may be the sign of underlying inherited thrombophilia [2]. However there was no history of lupus erythematosus, or thrombotic or hemorrhagic tendency in the mother, siblings, or other family members and coagulation studies of the patient were within normal ranges. The lesion slowly decreased in size and had almost fully resolved within 3 weeks. He was healthy and 9 months old at the time of writing this paper. The lesion may represent an epiphenomenon from an injury to cutaneous blood vessels, and our patient may be within the spectrum of Nicolau syndrome (NS). NS is a clinical presentation of livedoid dermatitis that occurs after intramuscular injection [3, 4]. However its pathogenesis is not well understood. The pathophysiology of NS involves intra-arterial and/or para-arterial injection and arterial embolism of viscous suspensions of drugs meant for intramuscular, intravenous; or intra-articular injection, followed by acute vasospasm, hence the livedo-like appearance or this was likely some other effect of the injection possibly related to the act or trauma of injection itself in an infant with very thin and easily traumatized skin [3, 4]. Histology shows thrombosis of small and medium sized vessels of the reticular dermis without signs of vasculitis [4]. The clinical manifestations of NS are pallor and pain occurring soon after injection followed by erythema that progresses to a livedo-like dermatitis and skin necrosis [4]. However, there was only a history of trauma as a result of the heel prick without viscous suspensions of drugs in our patient. We speculated that the lesion may be NS related to the trauma by the heel prick in our case. We previously reported a case of NS developing approximately 2 h after the intramuscular administration of vitamin K1 in an extremely low-birth-weight premature newborn [5]. Puvabanditsin et al. reported NS induced by intramuscular vitamin K injection in two extremely low-birth-weight infants [6]. Its pathogenesis is not well understood. There is no specific therapy for NS other than prevention [3]. 

 Although heel prick has been performed all over the world for many years, we could not find any reports in the English language literature on foot skin ischemic necrosis due to heel prick in neonates. This patient is, to our knowledge, the first case of foot skin ischemic necrosis due to heel prick in newborns.
